# 

**Published:** 1887-02

**Authors:** 


### BOOKS RECEIVED.

MANUAL OF OPERATIVE SURGERY. Bryant. D. Appleton & Co., New York. 1887.

TEXT-BOOK OF MEDICINE. Strümpell. D. Appleton & Co., New York. 1887.

PRACTICE OF MEDICINE. Bartholow. D. Appleton & Co., New York. 1886.

AN EPITOME OF THE NEWER MATERIA MEDICA. Parke, Davis & Co., Detroit. 1886.

MANUAL OF AUSCULTATION, ETC. Flint. Lea, Brothers & Co., Philadelphia.

DISEASES OF THE LUNGS AND PLEURÆ. Powell. Wm. Wood & Co., New York. 1886.

THE BLOOD, NUTRITION AND INFECTIOUS DISEASES. Eichharst. Wm. Wood & Co., New York. 1886.

OPERATIVE SURGERY. Smith. Lea, Brothers & Co., Philadelphia. 1887.

### OUR ADVERTISERS.

WHEN you want pure pepsin, specify Fairchild's.

SEE advertisement of the well-known Atlanta clothier, George Muse, on page 24.

SEE new advertisement of Johann Hoff's pure Extract of Malt, next to "Book Reviews," and if you need it send to your druggist for it. See that you get no imitation.

FONT HILL TRAINING SCHOOL.—This institution, situated in Ellicott City, Maryland, has an advertisement on page 35, to which we direct the attention of our readers.

THE New York Pharmacal Association, of Yonkers, N. Y., manufacturers of the well-known lactopeptine, have issued an interesting annual, which will be sent to any physician on application.

FAIRCHILD BROS. & FOSTER, NEW YORK.—By reference to the advertisement of this firm, to be found next to "Society Reports," our readers will find something of interest regarding rectal alimentation. This firm has been for a number of years paying special attention to the manufacture of digestive ferments and has succeeded in giving the profession pure and reliable goods. If you would have pure pepsin, in purchasing always specify Fairchild's.

COD LIVER OIL WITH HYPOPHOSPHITES.— Scott & Bowne, manufacturing chemists of New York, make a specialty of producing an emulsion of cod liver oil with hypophosphites. Their great care in selecting the oil and in making the combination is amply proven by the high therapeutical value set upon the emulsion by the profession. It is no new remedy, but has been steadily growing in demand for a number of years. It is certainly very useful in restoring wasting tissue, and in cases of scrofulous children it acts almost as a specific. They also offer a Buckthorn cordial which is highly useful in the treatment of constipation.—*Massachusetts Eclectic Medical Journal.*

SYRUPUS ROBORANS is one of the most efficient tonics and constructive agents that has been placed before the profession. One of its most commendable features is its palatableness. It is one of the few preparations containing phosphorous that is free from the very unpleasant taste of that most disagreeable drug.

SUBSTITUTION.—It is a well-known fact that there are druggists in every large city who are not to be trusted with the filling of a prescription that calls for any expensive drug. They come and go, so that at last physicians are compelled to designate certain of the drug fraternity as trustworthy, and insist upon their patients going to these alone for their medical supplies. If they fail to do this, their work is thrown away and their reputations go with the failure of their remedies in critical cases. The following case, from actual observation and experience, will illustrate this better than a volume of argument: Four ounces of a mixture of bromide of potassium and chloral, each an ounce, with tincture of hyoscyamus and fluid extract of cannabis indica, in appropriate doses, were ordered, with directions to take one teaspoonful every hour until sleep should be induced. An ugly, muddy mixture was received, which produced nausea and headache, but no sleep. A similar prescription, instead of the above extemporaneous officinal combination, was ordered; only "Battle's Bromidia" was designated, which induced refreshing sleep after a few doses of from twenty to thirty drops had been taken.—*Medical Brief, Prof. William B. Hazard.*

